# Bronchoscopic lesion resection combined with endobronchial instillation as a salvage therapy for a patient with complex aspergilloma complicated by non-tuberculous mycobacterial infection: case report

**DOI:** 10.3389/fmed.2026.1772474

**Published:** 2026-04-02

**Authors:** Xiaoni Zhou, Xiaohui Luo, Yuying Wu, Han Wang, Zhiyun Pan, Jun Wang, Xinhua Xiao, Zhi Yao

**Affiliations:** Department of Pulmonary and Critical Care Medicine, Wuhan Pulmonary Hospital, Wuhan, Hubei, China

**Keywords:** aspergilloma, bronchoscopy, liposomal amphotericin B, non-tuberculous mycobacteria, pulmonary aspergillosis

## Abstract

**Background:**

The infection rate of *Aspergillus* after non-tuberculous mycobacterial lung disease shows a persistent upward trend. While current treatment options for patients with complex pulmonary aspergillosis remains under investigation, the optimal treatment strategy remains under investigation.

**Case presentation:**

A 66-years-old female was diagnosed with non-tuberculous mycobacterial lung disease complicated by a complex pulmonary aspergilloma involving the posterior segment of the right upper lobe and the dorsal segment of the right lower lobe. Due to scattered lesions and multiple underlying diseases, surgical resection could not be performed. Instead, based on oral low-dose voriconazole systemic antifungal therapy of 100 mg twice daily, four sessions of bronchoscopic lesion resection with biopsy forceps, and endobronchial instillation of liposomal amphotericin B 10 mg were selected. Finally, the aspergilloma disappeared, and the patient’s symptoms were relieved.

**Conclusion:**

This case report suggests that for patients with complex pulmonary aspergilloma and non-tuberculous mycobacterial lung infection who are ineligible for surgery and cannot tolerate high-dose systemic antifungal therapy, bronchoscopic lesion resection combined with endobronchial instillation therapy may represent a feasible treatment option with potential clinical benefits. It also serves as a reminder to remain vigilant for the occurrence of aspergillosis following non-tuberculous mycobacterial lung disease.

## Introduction

Pulmonary aspergilloma is a pulmonary fungal disease caused by *Aspergillus* infection, characterized by the formation of non-invasive spherical mycelial masses from *Aspergillus* spores proliferating within old cavitary pulmonary lesions. Radiologically, it presents as a mobile, round or oval solid shadow (1). As one of the five subtypes of chronic pulmonary aspergillosis, pulmonary aspergilloma typically occurs in patients with structural lung lesions like bronchiectasis or tuberculous cavities (2). Approximately 50%–80% of patients present asymptomatically, often detected incidentally during imaging follow-up. Recurrent or massive hemoptysis represents its most severe complication, while some patients exhibit nonspecific clinical symptoms such as chronic cough, sputum production, and weight loss (3).

Although various treatment approaches exist for pulmonary aspergilloma, the optimal treatment strategy remains unclear, and its natural history has not been extensively studied. Approximately 10% of patients achieve spontaneous resolution without intervention. Whether to treat asymptomatic pulmonary aspergilloma remains controversial, primarily due to the relatively high mortality rate during the acute phase when massive hemoptysis occurs. Although the 2025 British Thoracic Society Clinical Statement on *Aspergillus*-related chronic lung disease recommends against routinely offering surgical intervention or antifungal therapy for asymptomatic aspergilloma (4). For symptomatic patients, surgical resection is the most effective treatment; however it is only applicable to simple aspergillomas and certain complex cases with focal lesions (5). Despite stringent surgical indications, postoperative risks remain substantial. Reports indicate a mortality rate of nearly 4% following surgical intervention for aspergilloma, alongside significant postoperative complication rates (6, 7). Bronchial artery embolization serves as a bridging measure for life-threatening hemoptysis, achieving an immediate hemostasis rate in over 80%. However, the recurrence rate within 1 year reaches as high as 50% (8). For patients with surgical contraindications, nonsurgical strategies mentioned in the 2015 European Society of Clinical Microbiology and Infectious Diseases (ESCMID) and the European Respiratory Society (ERS) guidelines on managing chronic pulmonary aspergillosis include systemic administration or nebulized inhalation of amphotericin B, long-term oral azole therapy. These guidelines also mention intracavitary local therapy for chronic pulmonary aspergillosis, such as antifungal instillation via bronchoscopy–guided endobronchial catheters, computed tomography (CT)–guided percutaneous transthoracic needles, or catheters directly placed into the aspergilloma cavity (9). Additionally, some researchers have proposed endobronchial instillation of antifungal agents (10). Unfortunately, these treatments remain uncertain, with reported clearance rates of aspergillomas varying considerably across studies (11). Therefore, the optimal management strategy for pulmonary aspergillomas requires further exploration.

*Mycobacterium tuberculosis* and non-tuberculous mycobacteria (NTM) infections are major risk factors for the development of chronic pulmonary aspergillosis (12). Since both NTM lung disease and pulmonary aspergillosis can present with chronic cough, sputum production, and even hemoptysis, early pulmonary aspergillosis infection is easily ignored during the course of NTM lung disease. Furthermore, while long-term multidrug chemotherapy is the core treatment for NTM lung disease, it severely compromises patients’ immune function. Even more troubling is the high recurrence rate of NTM infections, which brings great challenges to surgical intervention and conventional antifungal therapy in patients with concurrent pulmonary aspergillosis following NTM infection. Developing safe and effective management strategies for such patients has become an urgent priority.

With consideration of the increasing number of patients with complex pulmonary aspergillomas following *Mycobacterium tuberculosis* and NTM infections, as well as the ongoing challenges in managing such cases, we report a case of complex pulmonary aspergilloma coinfected with NTM treated with bronchoscopic staged lesion resection combined with endobronchial cavity instillation. The patient concurrently received systemic antifungal therapy with low-dose oral voriconazole.

## Case presentation

The patient provided informed consent, and this study was approved by the Ethics Committee of Wuhan Pulmonary Hospital. The completed CARE checklist is available as Supplementary material.

**FIGURE 1:**
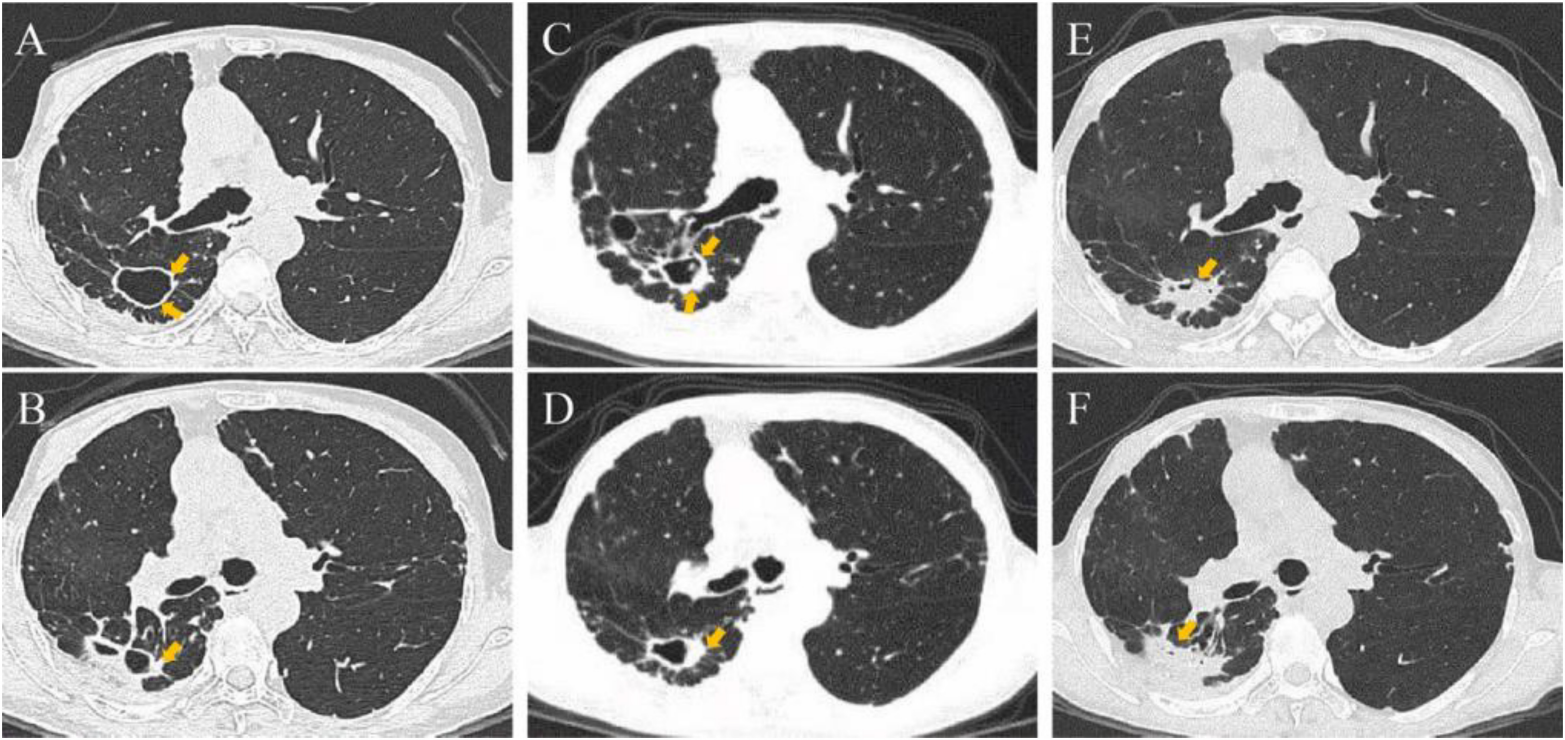
This patient’s follow-up chest CT images for suspected *Aspergillus* infection. **(A,B)** Multiple cavities remain in the right lung due to tuberculosis. **(C,D)** The cavity walls of the posterior segment of the right upper lobe and the dorsal segment of the right lower lobe thickened in December 2022. **(E,F)** Contents appeared in the cavities of the right lung in March 2023.

A 66-years-old female with a history of chronic obstructive pulmonary disease, diagnosed with pulmonary tuberculosis in 1991, underwent 1 year of standardized anti-tuberculosis treatment, leaving multiple thin-walled cavities in the right lung (Figures 1A,B). In 2012, she was diagnosed with breast cancer and treated with a radical mastectomy followed by four cycles of chemotherapy. In 2015 and 2019, she underwent bronchial artery embolization for massive hemoptysis. In 2021, she was diagnosed with NTM lung disease (*Mycobacterium avium* complex) and received anti-NTM therapy with azithromycin, ethambutol, rifampicin, and amikacin. Presenting to the respiratory department on December 28, 2022, with “fever accompanied by cough for 1 month,” a CT scan revealed partial thickening of the cavity wall in the posterior segment of the right upper lobe compared to previous imaging (Figures 1C,D). However, no further investigations were conducted, and NTM treatment was continued.

On March 27, 2023, she was hospitalized for a follow-up examination after 2 years of anti-NTM treatment. Chest CT revealed contents within cavities in the posterior segment of the right upper lobe and the dorsal segment of the right lower lobe (Figures 1E,F). Bronchoscopy showed no significant abnormalities. Bronchoalveolar lavage fluid (BALF) fungal smear and culture were negative. Mycobacterial culture, *Mycobacterium tuberculosis* DNA testing, and NTM species identification were all negative. Given the absence of pathogenetic evidence for *Aspergillus* infection, antifungal therapy was not initiated. Considering the patient’s negative NTM culture conversion and completion of the full treatment course, anti-NTM therapy was discontinued.

**FIGURE 2:**
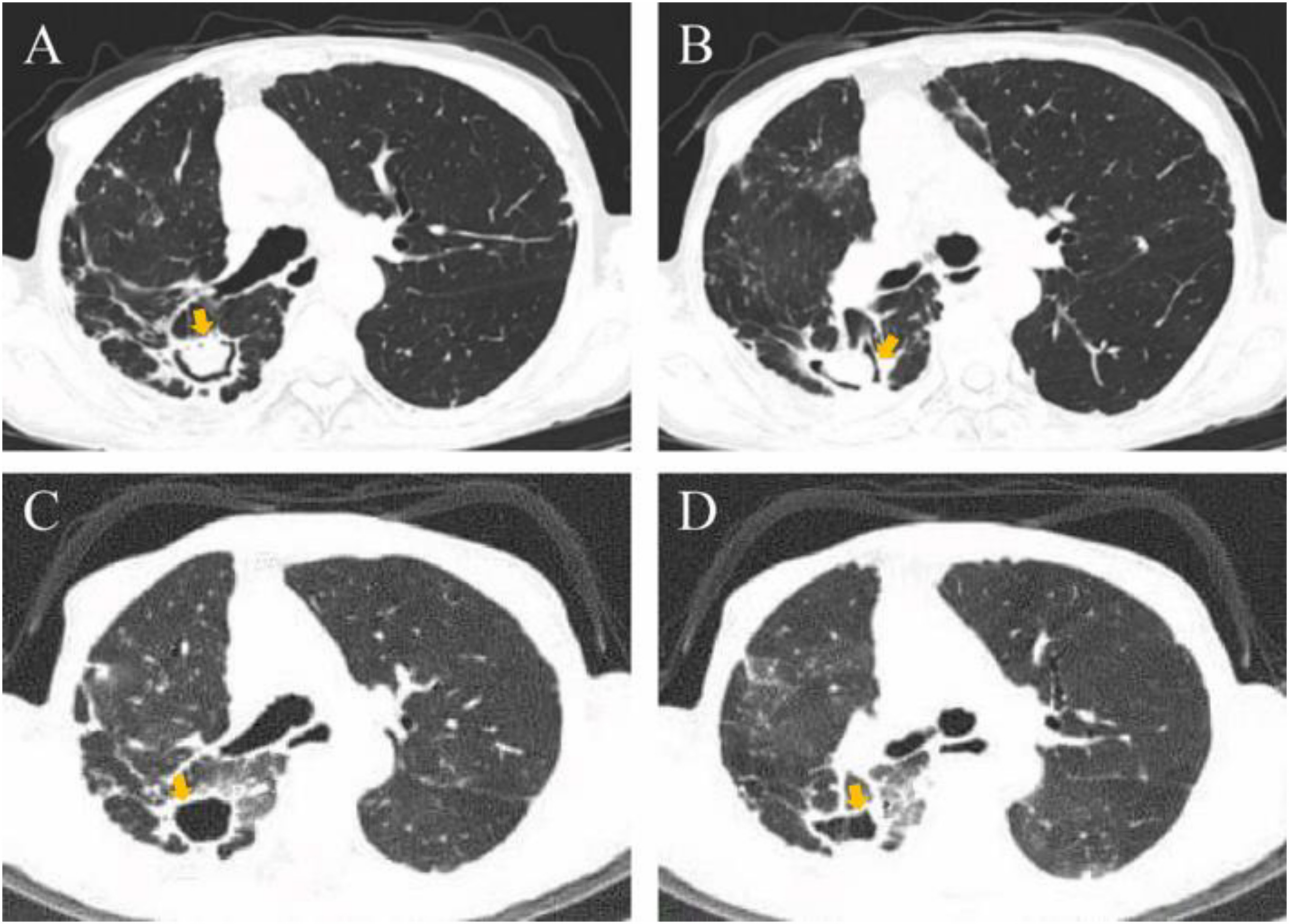
Chest CT images of the patient before and after bronchoscopy. **(A,B)** The manifestation of *Aspergillus* ball in the posterior segment of the right upper lobe and the dorsal segment of the right lower lobe before bronchoscopy in July 2025. **(C,D)** The *Aspergillus* ball disappeared after the bronchoscopy operation.

**FIGURE 3:**
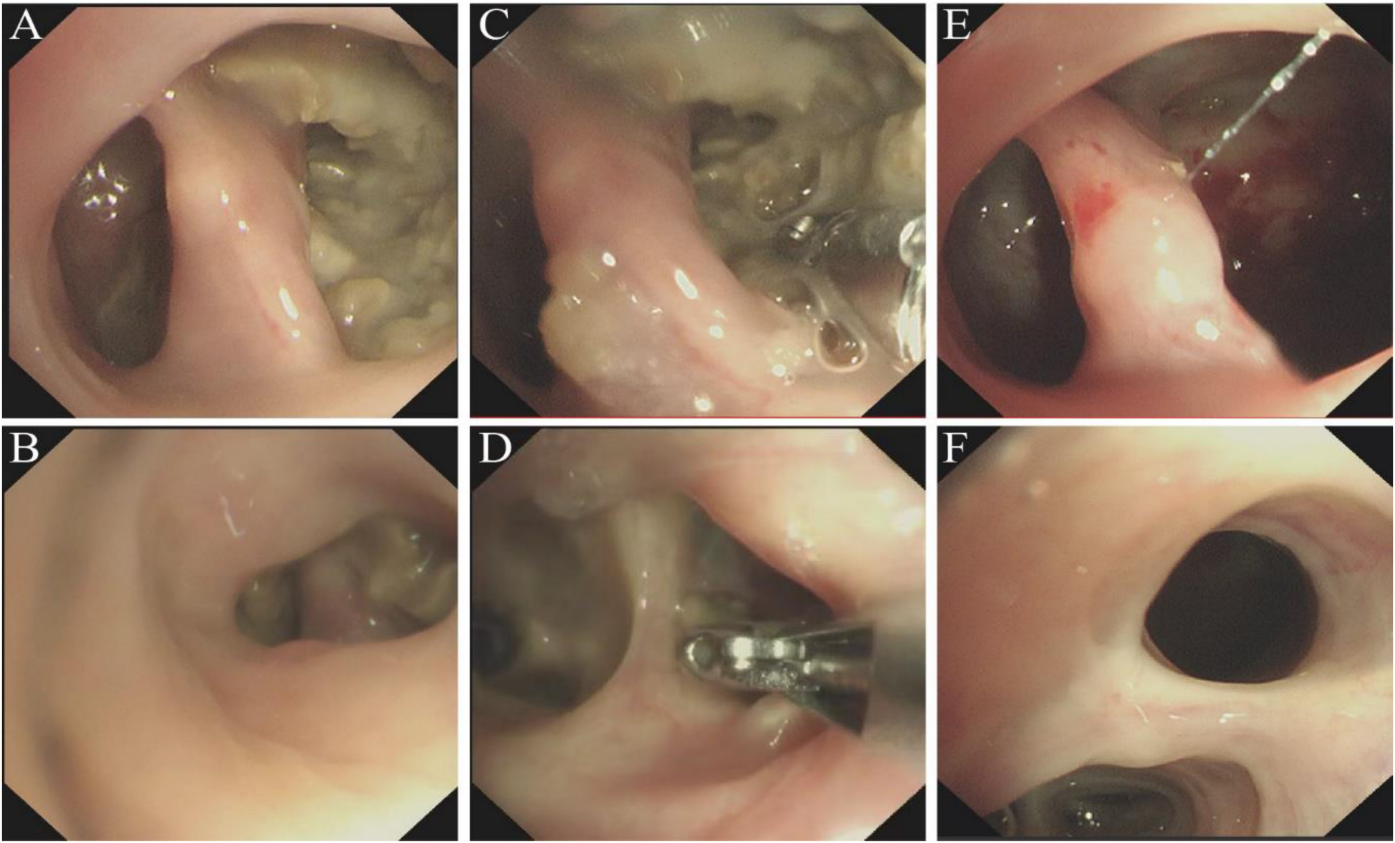
Bronchoscopy image showing an *Aspergillus* ball in the posterior segment of the right upper lobe and the dorsal segment of the right lower lobe. **(A,B)** After the mucus was suctioned, a coral-like shape was observed. **(C,D)** The aspergilloma was then removed with forceps. **(E,F)** The blind ended bronchus after removal.

**FIGURE 4:**
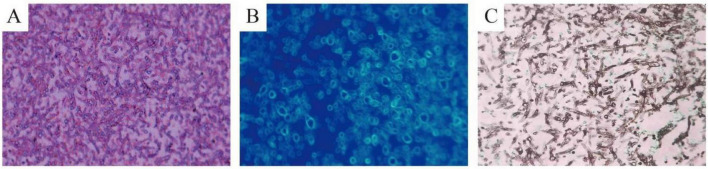
Pathological findings of *Aspergillus fumigatus*. **(A)** Paraffin-embedded tissue section with hematoxylin and eosin staining. **(B)** Paraffin-embedded tissue section with immunofluorescence staining. **(C)** Paraffin-embedded tissue section with Gomori methenamine silver stain.

On July 10, 2025, she was hospitalized again for “intermittent cough with sputum production and hemoptysis for 1 week.” Physical examination revealed a temperature of 36.7 °C (97.9°F) and no abnormal breath sounds heard in the lungs. Laboratory test results were as follows: serum C-reactive protein at 1.89 mg/dL, white blood cell count at 4400/μL, both liver and kidney function indicators within normal ranges, negative β-D-glucan test and *Aspergillus* galactomannan ELISA, with anti-*Aspergillus* immunoglobulin G (IgG), > 80 AU/mL (Cutoff: <80 AU/mL is negative, 80–120 AU/mL is weakly positive, >120 AU/mL is positive; Test kit provided by Tianjin Danna Biotechnology Co., Ltd.). A repeat chest CT scan found cavitary lesions with intraluminal nodules in the posterior segment of the right upper lobe and the dorsal segment of the right lower lobe, presenting a classic “fungal ball” appearance (Figures 2A,B). Additionally, the two cavities were interconnected. Bronchoscopy showed mucus-covered *Aspergillus* balls within the lumens of various segments of the posterior segment of the right upper lobe and the A segment of the dorsal segment of the right lower lobe. After mucus suction, a coral-like structure was exposed (Figures 3A,B). Subsequent repeated attempts at debridement using biopsy forceps were performed (Figures 3C,D). However, due to excessive necrotic material within the lumens, the patient’s sedation status, and prolonged procedure duration, we ultimately decided to conduct staged debridement after assessing the bleeding risk. Postoperative tissue culture and histopathology of BALF both demonstrated *Aspergillus fumigatus* (Figures 4A–C), leading to a final diagnosis of aspergilloma with chronic progressive pulmonary aspergillosis.

Meanwhile, her BALF mycobacterial culture was positive again, with NTM species identification indicating *Mycobacterium abscessus*. Considering the patient’s imaging findings did not correlate with *Mycobacterium abscessus* infection, and the strain did not match her previous mycobacterial infections, we decided to withhold anti-*Mycobacterium abscessus* therapy. Instead, on July 15, 2025, we initiated amphotericin B nebulization at 10 mg/day. Regarding oral antifungal agents, considering that the patient could not afford posaconazole or isavuconazole, we selected voriconazole from the first-line drugs. Given the patient’s weight of only 33 kg, voriconazole oral therapy was administered at 100 mg twice daily. Subsequently, we performed bronchoscopic lesion debridement on July 23 and July 31, administering 10 mg of liposomal amphotericin B intrabronchially during each procedure. The patient’s cough and sputum production improved significantly, and hemoptysis ceased. For safety reasons, we decided to discharge her for 2 weeks of home rest while continuing oral voriconazole antifungal therapy.

On August 20, 2025, she returned to the hospital to continue her follow-up treatment. The fourth bronchoscopy showed a significant reduction in coral-like necrotic material within the bronchial lumen. We continued the previous treatment plan, combining lesion resection with endoscopic medication delivery. Encouragingly, the final bronchoscopy on August 28 demonstrated complete resolution of necrotic material within the bronchial lumen (Figures 3E,F). Throughout the entire course, she experienced no adverse reactions such as postoperative hemoptysis or hypoxemia. Postoperative chest CT revealed the disappearance of the *Aspergillus* ball within the cavities of the posterior segment of the right upper lobe and dorsal segment of the right lower lobe (Figures 2C,D). Surprisingly, follow-up BALF culture yielded negative results for NTM species. A comprehensive evaluation suggests a high likelihood of *Mycobacterium abscessus* colonization in the patient’s lung; however, definitive confirmation awaits the results of mycobacterial culture. The patient’s cough and sputum production have now nearly ceased. She continues a full course of oral voriconazole for antifungal therapy. Given the high recurrence rate of pulmonary aspergilloma, we still recommend regular bronchoscopic drug delivery therapy. Three months later, the patient returned for a follow-up examination. Chest CT revealed that the original cavity wall remained smooth with no signs of recurrence of the aspergilloma. Unfortunately, the patient’s previous mycobacterial culture was positive, and the species was identified as *Mycobacterium intracellulare*. This matched the NTM species identified in the BALF during this follow-up examination. Consequently, we must consider this a recurrence of NTM infection.

## Discussion and conclusions

Pulmonary aspergilloma is a relatively common clinical subtype of chronic pulmonary aspergillosis; the incidence of pulmonary *Aspergillus* co-infection has been increasing due to the rising prevalence of NTM. Existing studies suggest an incidence rate ranging from 3.9% to 16.7% (13, 14). Within this subtype, approximately 56%–78.3% of cases present not with simple aspergillomas but with complex pulmonary aspergillomas (5, 15), rendering surgical intervention no longer the optimal choice for most patients. In recent years, discussions on treatment strategies for pulmonary aspergilloma have been numerous, yet therapeutic outcomes remain suboptimal.

In this case report, we describe the clinical management of a 66-years-old female patient diagnosed with NTM lung disease who developed pulmonary aspergillosis during NTM treatment. Due to the insidious and nonspecific clinical manifestations of pulmonary aspergillosis, she did not receive an appropriate diagnosis and treatment in the early stages until her clinical symptoms worsened. Given the patient’s history of tuberculosis and breast cancer, combined with NTM lung disease, we determined that surgical resection of the aspergilloma was not suitable. Additionally, her thin physique precluded tolerance of high-dose oral voriconazole antifungal therapy. Therefore, while administering systemic treatment with low-dose oral voriconazole, we employed a combined approach for complex pulmonary aspergilloma: bronchoscopic lesion resection combined with endobronchial intra-lesional injection of liposomal amphotericin B. This treatment ultimately resolved the aspergilloma and led to the patient’s recovery.

Previously, researchers have also proposed treating symptomatic pulmonary aspergillomas with bronchoscopic lesion debridement, particularly for patients ineligible for surgical resection (16, 17). Dumoulin et al. recommended a multimodal treatment strategy combining “pretreatment + consolidation” antifungal therapy with endoscopic debridement as an alternative for non-surgical patients. They found that approximately 94% of patients achieved significant clinical and radiographic improvement during follow-up. Additionally, all patients in this study received oral voriconazole 300 mg twice daily (18). However, not all patients can tolerate high-dose voriconazole maintenance therapy. How can they achieve effective treatment when faced with extremely high *Aspergillus* loads? Bronchoscopic cavity-directed drug instillation serves as a highly effective adjunctive local therapy for pulmonary aspergilloma (19). Local administration achieves high drug concentrations within cavities, directly eradicating *Aspergillus* fungi. However, when used alone as adjunctive therapy to systemic treatment, this approach requires prolonged treatment cycles. For patients requiring urgent clinical symptom improvement, this strategy is unsuitable. In this case report of a complex pulmonary aspergilloma, where the patient could only tolerate low-dose voriconazole systemic therapy, we adopted a combined management strategy of bronchoscopic lesion resection plus endoscopic cavity drug instillation. This approach led to the rapid resolution of the aspergilloma and partially compensated for the limitations of systemic therapy.

On the other hand, studies indicate that underlying NTM lung disease is an independent poor prognostic factor for chronic pulmonary aspergillosis (20). Due to the high rate of misdiagnosis, coupled with drug interactions between treatments for the two conditions, patients face a significantly worse prognosis. Therefore, early identification and prompt treatment of aspergillosis are crucial during anti-NTM therapy for patients with NTM lung disease. The ESCMID, the ERS, and the European Consortium for Medical Mycology (ECMM) jointly established diagnostic criteria for chronic pulmonary aspergillosis, including: (i) chest imaging findings (preferably CT) persisting for ≥3 months, (ii) direct evidence of *Aspergillus* infection or an immunological response to *Aspergillus* species, and (iii) exclusion of other diagnoses (9). Microbiological and immunological evidence is essential for diagnosing chronic pulmonary aspergillosis, though their positivity rates vary depending on the individual and disease stage. *Aspergillus*-specific IgG and IgE antibodies hold diagnostic value for chronic pulmonary aspergillosis (21, 22). Studies indicate that 30%–32% of pulmonary aspergillosis patients exhibit elevated serum levels of anti-*Aspergillus* IgG (23, 24). Additionally, *Aspergillus* polymerase chain reaction testing of respiratory secretions holds significance in the diagnosis and management of pulmonary aspergillosis, while serum galactomannan testing demonstrates higher specificity for invasive aspergillosis. Given that NTM infection is a significant risk factor for the development of pulmonary aspergillosis, we should pay close attention to the occurrence of aspergillosis following NTM infection. When imaging or clinical symptoms strongly suggest pulmonary aspergillosis, enhanced diagnostic measures are recommended to improve detection rates. Furthermore, these patients warrant heightened monitoring during follow-up.

In summary, this study aims to assist clinicians in managing complex pulmonary aspergilloma cases where full-dose systemic antifungal therapy is contraindicated by employing a combined treatment strategy of bronchoscopic lesion resection plus endoscopic cavity-directed drug delivery. Furthermore, through this case, we wish to alert clinicians to the potential for aspergillosis infection following NTM lung disease, thereby securing additional time and treatment options for managing the condition. A limitation of this study is that bronchoscopic lesion resection is only suitable for patients with accessible lesions and does not apply to all complex pulmonary aspergillomas. However, for cases where the procedure is feasible, this combined therapeutic approach may represent a viable option worth promoting in the absence of an optimal treatment for pulmonary aspergilloma. We hope future large-scale studies will validate these findings.

## Data Availability

The original contributions presented in this study are included in this article/Supplementary material, further inquiries can be directed to the corresponding author.
