# Supplementary material for: Bronchoscopic lesion resection combined with endobronchial instillation as a salvage therapy for a patient with complex aspergilloma complicated by non-tuberculous mycobacterial infection: case report
Source: Front Med (Lausanne). 2026 Apr 2;13:1772474. doi: 10.3389/fmed.2026.1772474 (PMC13083207; doi:10.3389/fmed.2026.1772474)
Supplement: Supplementary file 1 [file Data_Sheet_1.pdf]

CARE Checklist of information to include when writing a case report

CARE case report guidelines

(cc) BY-NC-ND

Topic	Item	Checklist item description	Reported on Line
Title	1	The diagnosis or intervention of primary focus followed by the words "case report"	Title page,page 1
Key Words	2	2 to 5 key words that identify diagnoses or interventions in this case report, including "case report"	Keywords, page 2
Abstract (no references)	3a	Introduction: What is unique about this case and what does it add to the scientific literature?	Abstract, paragraph 1, page 2
	3b	Main symptoms and/or important clinical findings	Abstract, paragraph 2, page 2
	3c	The main diagnoses, therapeutic interventions, and outcomes	Abstract, paragraph 2, page 2
	3d	Conclusion—What is the main "take-away" lesson(s) from this case?	Abstract, paragraph 3, page 2
Introduction	4	One or two paragraphs summarizing why this case is unique (**may include references**)	Introduction, page 2-3
Patient Information	5a	De-identified patient specific information.	Case presentation, page 4, paragraph 1
	5b	Primary concerns and symptoms of the patient.	Case presentation, page 4, paragraph 1
	5c	Medical, family, and psycho-social history including relevant genetic information	Case presentation, page 4, paragraph 1
	5d	Relevant past interventions with outcomes	Case presentation, page 4, paragraph 2
Clinical Findings	6	Describe significant physical examination (PE) and important clinical findings.	Case presentation, page 5-7
Timeline	7	Historical and current information from this episode of care organized as a timeline	N/A
Diagnostic Assessment	8a	Diagnostic testing (such as PE, laboratory testing, imaging, surveys).	Case presentation, page 5-7
	8b	Diagnostic challenges (such as access to testing, financial, or cultural)	Case presentation, page 5-7
	8c	Diagnosis (including other diagnoses considered)	Case presentation, page 6
	8d	Prognosis (such as staging in oncology) where applicable	Case presentation, page 7
Therapeutic Intervention	9a	Types of therapeutic intervention (such as pharmacologic, surgical, preventive, self-care)	Case presentation, page 6-7
	9b	Administration of therapeutic intervention (such as dosage, strength, duration)	Case presentation, page 6-7
	9c	Changes in therapeutic intervention (with rationale)	N/A
Follow-up and Outcomes	10a	Clinician and patient-assessed outcomes (if available)	N/A
	10b	Important follow-up diagnostic and other test results	N/A
	10c	Intervention adherence and tolerability (How was this assessed?)	Case presentation, page 5
	10d	Adverse and unanticipated events	N/A
Discussion	11a	A scientific discussion of the strengths AND limitations associated with this case report	Discussion and conclusion,, page 7-9
	11b	Discussion of the relevant medical literature **with references**.	Discussion and conclusion, paragraph 3-4, page 8-9
	11c	The scientific rationale for any conclusions (including assessment of possible causes)	Discussion and conclusion, page 7-9
	11d	The primary "take-away" lessons of this case report (without references) in a one paragraph conclusion	Discussion and conclusion, paragraph 5, page 9
Patient Perspective	12	The patient should share their perspective in one to two paragraphs on the treatment(s) they received	N/A
Informed Consent	13	Did the patient give informed consent? Please provide if requested	Yes ☑ No ☐
